# Effects of an 8-week minimal-dose home-based eccentric exercise program on physical health and exercise adherence

**DOI:** 10.1007/s00421-025-05989-7

**Published:** 2025-10-09

**Authors:** Benjamin J. C. Kirk, Georgios Mavropalias, Anthony J. Blazevich, Jodie L. Cochrane Wilkie, Aus Molan, Kazunori Nosaka

**Affiliations:** 1 School of Medical and Health Sciences, Edith Cowan University, Joondalup, WA Australia; 2 Physical Activity, Sport and Exercise Research Theme, Faculty of Health, Southern Cross University, Gold Coast, QLD Australia; 3 PathWest Laboratory Medicine, Nedlands, WA Australia; 4 Exercise Medicine Research Institute, Edith Cowan University, Joondalup, WA Australia; 5 Performance and Expertise Research Centre, Macquarie University, Sydney, NSW Australia; 6 Biomechanics, Physical Performance, and Exercise Research Group, Macquarie University, Sydney, NSW Australia

**Keywords:** Eccentric exercise, Physical fitness, Exercise adherence, Mental well-being, Health behavior change, Minimal-dose exercise

## Abstract

**Purpose:**

This study investigated whether extending a previously tested minimal-dose 4-week, 5-min daily home-based eccentric exercise program to 8 weeks would lead to continued improvements in physical fitness, health markers, and mental well-being in sedentary individuals, and whether it could promote sustained exercise habits up to 12 months post-intervention.

**Methods:**

Ten sedentary participants (54 ± 9 y) completed an 8-week daily home-based exercise program involving four bodyweight-based exercises (chair squats, wall push-ups, chair-reclines, and heel drops) and their progressed variations. Outcome measures were collected at baseline and 4 and 8 weeks, including isometric mid-thigh pull (IMTP), handgrip strength (HG), push-ups, sit-ups, sit-and-reach (S&R), body composition, blood markers, and mental well-being (SF-36 and Subjective Vitality Scales [SVS]). Exercise adherence was calculated from daily exercise logs. Physical activity engagement was assessed via follow-up surveys at 1, 3, and 12 months post-intervention.

**Results:**

Adherence remained high (weeks 1–4: 94 ± 11%; weeks 5–8: 93 ± 11%). IMTP (7.3 ± 12.2%), push-ups (19.5 ± 18.2%), sit-ups (28.5 ± 44.8%), and S&R (7.6 ± 13.6%) further improved (*p* < 0.05) in weeks 5–8 but gains were attenuated relative to weeks 1–4. No significant changes were observed in HG, body composition, or blood markers. SF-36 improved (31.9 ± 56.3%, *p* < 0.05) during weeks 1–4 only. At 12-month follow-up, 90% of participants reported ongoing physical activity.

**Conclusion:**

Extending a low-dose, home-based exercise program to 8 weeks led to continued, though attenuated, improvements in physical fitness, with mental well-being benefits emerging early. High adherence and sustained activity at follow-up suggest this minimal-dose intervention may support lasting exercise behavior change in sedentary adults.

**Supplementary Information:**

The online version contains supplementary material available at 10.1007/s00421-025-05989-7.

## Introduction

Physical inactivity is a leading modifiable risk factor for chronic disease and premature mortality, estimated to contribute to over 5 million annual deaths globally (Lee et al. 2012). Despite widespread public health efforts, a large proportion of adults still fail to meet the minimum recommended activity levels (Australian Institute of Health and Welfare 2023; Bennie et al. 2015). Regular exercise offers well-established benefits for cardiorespiratory fitness, muscular strength, metabolic health and mental well-being (Garber et al. 2011; Mahindru et al. 2023), yet sustaining participation remains a significant challenge for many individuals.

One of the most frequently cited barriers to regular exercises is a perceived lack of time (Bowles et al. 2002; Hoare et al. 2017), underscoring the need for accessible, time-efficient interventions that can be easily integrated into daily life. Emerging research has shown that minimal-dose exercise programs can lead to meaningful improvements in physical fitness, body composition, and mental well-being (Yoshida et al. 2024; Katsura et al. 2019; Morgan et al. 2013; Nuzzo et al. 2024). In our previous study (Kirk et al. 2025), a 5-min daily home-based eccentric contraction-focused exercise program comprising body-weight chair squats, chair reclines, wall push-ups, and heel drops (10 repetitions each with progressions), produced significant short-term benefits. After 4 weeks, sedentary individuals demonstrated improvements in muscle strength (13%), flexibility (9%), strength endurance (sit-ups: 51%; push-ups: 66%), and mental health (SF-36: 16%; Subjective Vitality Scale: 20%), alongside high adherence (89%). These findings highlight the potential of brief, home-based interventions to mitigate the health risks associated with sedentary behavior.

However, the long-term sustainability of these benefits requires further investigation. Physiological adaptations such as muscle hypertrophy, improved cardiovascular efficiency, and beneficial metabolic shifts typically necessitate extended periods of regular exercise (Hughes et al. 2018). Moreover, while mental health improvements were evident in the short term, the persistence or amplification of these benefits over a more extended period remains uncertain. In our previous study (Kirk et al. 2025), no statistically significant group-level changes were observed in blood biomarkers over time. However, we noted that some participants who initially presented with values outside the clinical reference ranges returned to within those limits after 4  weeks. For example, one participant improved in each of cholesterol, low-density lipoprotein (LDL), high-density lipoprotein (HDL), and triglycerides, while two participants showed normalization in high-sensitivity C-reactive protein (hsCRP) levels. Although these individual changes were not the primary focus of the earlier analysis, they suggest that certain metabolic improvements may occur on a case-by-case basis and could become more pronounced with a longer intervention or increased training stimulus. Thus, identifying optimal exercise intensity, volume, and progression strategies for sustained long-term health improvements warrants further exploration.

Maintaining long-term adherence to exercise presents notable challenges, with approximately half of new exercisers reverting to sedentary or less active behaviors within a few months (Dishman and Buckworth 2013; Marcus et al. 2000). Understanding the factors that influence sustained exercise participation is crucial for developing effective public health strategies. Although our previous study provided valuable insights into short-term integration of exercise into daily routines, extending the intervention duration could reveal additional barriers and facilitators affecting long-term adherence.

To build upon our prior findings (Kirk et al. 2025), the present study extended the previously tested 4-week home-based bodyweight eccentric exercise program to 8 weeks, allowing for a more comprehensive evaluation of its longer-term effects on physical fitness, health markers, and mental well-being in previously sedentary individuals. Given the continuity in study design and participant cohort, this extension enabled further exploration of how minimal-dose exercise might support sustained physiological and psychological benefits. In addition, exercise adherence and behavior were tracked for up to 12 months post-intervention to assess the feasibility of fostering long-term exercise habits. The findings contribute to the growing body of evidence supporting brief, practical home-based exercise programs as effective strategies to counteract the health risks of sedentary lifestyles.

## Methods

### Participants

Following the completion of the initial 4-week intervention described previously (Kirk et al. 2025), a subset of 13 participants volunteered to continue the exercise program for an additional 4 weeks. This extension was offered in response to strong participant interest in continuing, although only a portion of the original cohort was eligible or available to proceed, as some had completed the initial study earlier and begun other programs. All participants were previously classified as inactive or minimally active using the international physical activity questionnaire, with none engaged in structured exercise at baseline. In the present study, the term “sedentary” is used to describe individuals not meeting recommended moderate-to-vigorous physical activity guideline (Bull et al. 2020).

Three participants withdrew during the extended intervention due to illness (e.g., influenza) or unrelated health issues, resulting in 10 participants completing the full 8-week protocol. The final cohort (2 men, 8 women) had a mean ± standard deviation (SD) age of 54 ± 9 years (range: 40–69), height of 168.1 ± 8.0 cm (157–182), and body mass of 79.9 ± 14.3 kg (50.9–104.5). All participants provided written informed consent and confirmed they were physically capable of completing the study procedures. Ethical approval was granted by the Edith Cowan University Human Research Ethics Committee (REMS No: 2021-03011-KIRK), and all procedures were conducted in accordance with the Declaration of Helsinki.

### Study design

Participants continued the home-based body-weight eccentric exercise program for an additional 4 weeks beyond the previously completed 4-week intervention, resulting in a total of 8 weeks of training. In addition to the original timepoints (baseline, after a 2-week control period, and 4 weeks), participants were also assessed at 8 weeks following the extended intervention. The changes from weeks 5 to 8 were then compared to those observed during weeks 1–4. Post-training testing occurred in the morning 1 to 2 days following the final exercise session. Participants arrived in a fasted state for blood sampling and body composition assessment.

Each participant was provided with a Fitbit Charge 5 device (Fitbit Inc., San Francisco, CA, USA) to wear for the duration of the 8-week intervention. A unique email account was created for each participant to allow the investigator to remotely track daily step counts via the Fitbit user interface app. The participants were instructed to maintain their usual physical activity and dietary habits throughout the intervention aside from the prescribed exercise program.

### Exercise program

Each day, participants completed four eccentric-biased bodyweight exercises targeting different muscle groups, beginning with chair squats (lower-body push), wall push-ups (upper-body push), chair reclines (abdominals), and heel drops (calves), with more challenging variations introduced as needed. Eccentric exercise has been shown to improve muscle strength (Roig et al. 2009; Schoenfeld et al. 2017) and flexibility (Kay et al. 2023; Diong et al. 2022), with lower cardiovascular strain and perceived effort compared to concentric-focused or traditional resistance training (Hollander et al. 2003; Miller et al. 2009; Vallejo et al. 2006). These characteristics make eccentric-biased exercise particularly suitable for previously sedentary individuals, as the reduced effort and strain may help lower barriers to initiating and sustaining regular exercise participation. Participants performed one exercise from each category for a target of 10–20 repetitions, stopping when perceived exertion reached approximately 7 out of 10 on the rating of perceived exertion (RPE) scale (i.e., challenging but not maximal effort); however, some participants exceeded this range if they had not yet reached the target effort level. Each repetition involved a controlled 5 s eccentric (lowering) and a 1 s concentric (lifting) phase. Exercises could be performed in one session or distributed across the day to support flexibility and adherence. The program exercises and progression options are detailed in Online Resource 1.

In the initial 4-week phase, several participants progressed to more challenging exercise variations (e.g., pistol squats, V-ups) once 10 repetitions became easy (e.g., RPE < 5). However, in some cases the next exercise variation represented too large a jump in difficulty, making it difficult to sustain progression. To support more appropriate progressions and maintain training stimulus during the second 4-week block (weeks 5–8), participants were encouraged to advance to the next exercise level once they could complete 20 repetitions at an RPE of ≤ 7 on two consecutive days for a given movement. Progression criteria, exercise variations, and participant adherence data are summarized in Table 1. Participant exercise logs were maintained using secure, password-protected online spreadsheets, which were monitored daily by the lead investigator to verify compliance and progression.

**Table 1:** Summary of exercise adherence and progression for participants in different exercise categories over the 8-week intervention

Exercise category	Progression	Exercise	Weeks 1–4			Weeks 5–8				Participants finished at exercise (*n*)
			Participants completed (*n*)	Sessions completed	RPE	Participants completed (*n*)	Sessions completed	Repetitions	RPE	
Legs	1	Chair Squat	9	12 ± 11 [1–28]	6.1 ± 1.1 [4.0–7.2]	3	12 ± 14 [2–28]	14 ± 3 [10–16]	7.0 ± 1.0 [6.0–8.0]	1
	2	One-leg chair squat	7	17 ± 12 [5–28]	5.9 ± 1.8 [3.0–7.3]	6	20 ± 8 [7–28]	21 ± 16 [10–43]	6.1 ± 1.8 [3.0–7.8]	5
	3	Pistol squat	1	1 ± 1 [2–2]	7.0 ± 0.0 [7.0–7.0]	1	21 ± 0 [21–21]	10 ± 0 [10–10]	2.3 ± 0.0 [2.3–2.3]	1
Chest	1	Wall push-up	8	11 ± 11 [1–28]	6.2 ± 1.2 [4.0–7.0]	2	25 ± 5 [21–28]	23 ± 11 [15–30]	7.0 ± 0.0 [7.0–7.0]	1
	2	One-arm wall push-up	2	1 ± 0 [1–1]	10 ± 0.0 [10.0–10.0]	0	0 ± 0 [0–0]	0 ± 0 [0–0]	0 ± 0 [0–0]	0
	3	Table push-up	2	16 ± 16 [4–27]	7.5 ± 0.7 [7.0–8.0]	1	21 ± 0 [21–21]	10 ± 0 [10–10]	7.9 ± 0.0 [7.9–7.9]	1
	4	Knee push-up	5	22 ± 3 [17–25]	7.5 ± 0.7 [7.0–8.5]	3	19 ± 11 [7–27]	13 ± 3 [11–16]	7.3 ± 0.9 [6.6–8.3]	3
	5	Push-up	2	17 ± 16 [6–28]	3.0 ± 0.0 [3.0–3.0]	2	28 ± 0 [28–28]	15 ± 7 [10–20]	3.0 ± 0.0 [3.0–3.0]	2
Abdominals	1	Chair recline back	9	14 ± 12 [1–28]	6.5 ± 1.1 [4.5–7.6]	2	27 ± 2 [25–28]	13 ± 3 [11–15]	6.7 ± 0.4 [6.5–7.0]	2
	2	Chair Recline Back (legs straight)	5	15 ± 10 [1–27]	6.0 ± 2.0 [3.0–8.3]	1	12 ± 0 [12–12]	10 ± 0 [10–10]	–	0
	3	Sit-up	4	16 ± 9 [4–24]	6.1 ± 2.1 [3.0–7.6]	5	23 ± 5 [16–28]	15 ± 6 [10–24]	6.2 ± 2.2 [3.0–8.0]	5
Calves	1	Heel drop	3	19 ± 11 [6–28]	6.9 ± 0.1 [6.8–7.0]	3	19 ± 12 [5–28]	12 ± 3 [10–15]	7.1 ± 0.1 [7.0–7.2]	1
	2	Heel drop overstretch	9	23 ± 7 [6–28]	6.5 ± 1.3 [3.0–7.3]	6	21 ± 9 [4–28]	16 ± 11 [10–39]	5.9 ± 1.7 [3.0–7.2]	6
	3	1-leg heel drop overstretch	0	0 ± 0 [0–0]	0 ± 0 [0–0]	0	0 ± 0 [0–0]	0 ± 0 [0–0]	0 ± 0 [0–0]	0

Participants completed (*n*) indicates the number of participants who performed each exercise during the respective 4-week time blocks. “Sessions completed” and “RPE” are reported separately for weeks 1–4 and weeks 5–8 to capture progression and consistency over time. “Repetitions” reflect the number completed per set during weeks 5–8. All sets during weeks 1–4 were performed to a fixed target of 10 repetitions. “Participants finished at exercise (*n*)” denotes the number of participants for whom the listed exercise was the highest level attempted or maintained by the end of week 8. Exercises are listed in order of increasing difficulty within each category. RPE: rate of perceived exertion. Data are presented as mean ± SD (range). A dash (–) indicates data for that category was not reported

### Outcome measures

#### Anthropometric and body composition assessments

Anthropometric measures included height and body mass using a stadiometer and a calibrated scale, respectively. Lean body mass, fat mass, and body fat percentage were assessed using whole-body dual-energy X-ray absorptiometry (DEXA; Hologic Discovery X, USA). Participants were positioned supine on the scanner bed with limbs fully within the scanning area. Following the DEXA assessment, resting heart rate (HR), systolic blood pressure (SBP), and diastolic blood pressure (DBP) were measured using an automated sphygmomanometer (HEM-7122; Omron Healthcare Co., Ltd, Japan).

#### Physical fitness tests

Physical fitness was evaluated using a battery of tests: squat jump (SJ), countermovement jump (CMJ), isometric mid-thigh pull (IMTP), handgrip, push-up and sit-up endurance (maximum repetitions), and a 3-min step-up test. Jump performance was assessed on a force platform (Pasport Force Platform PS-2141; Pasco Scientific, USA) following standardized protocols, with participants performing three attempts for each jump and the best score used for analysis. The IMTP was conducted on a portable rig (VALD, Newstead, Australia) positioned over Pasport force plates (Pasco, Roseville, USA), with participants performing three maximal pulls separated by 1 min rest and the average score used for analysis. Handgrip strength was performed using a Jamar Smart Hand Dynamometer (Patterson Medical, IL, USA). Three attempts were performed on each hand with the best score of each hand used for analysis.

Push-up endurance was assessed using standardized technique criteria for men (toe push-ups) and women (knee push-ups), with invalid repetitions excluded. Sit-up performance was measured to a cadence (one repetition every 3 s). The test was terminated if cadence was not maintained for three consecutive repetitions. Flexibility was assessed using the sit-and-reach (S&R) test (Figure Finder Flex Tester, Novel Products Inc., USA) with the best of three attempts recorded. Cardiovascular fitness was evaluated via a 3 min step-up test (24 steps/min) using sex-specific box heights scaled to participant height. Heart rate (Polar H10, Polar Electro, Finland) and RPE (1–10 scale) were recorded at rest (HR_pre_), then at 5 s (HR_post_) and 1 min (HR_post,1-min_) following test completion.

#### Blood markers

Fasting blood samples were collected in the morning (after 10–12 h fasting) from the antecubital vein using vacutainers (ethylenediaminetetraacetic acid (EDTA), serum separating tubes (SST), plasma preparation tube (PPT), and fluoride/oxalate). Samples were processed and analyzed by PathWest Laboratory Medicine WA using accredited clinical chemistry and immunoassay procedures. Analytes included: HbA1c, glucose, insulin, fructosamine, triglycerides, total cholesterol, LDL, HDL, hsCRP, procollagen type 1 N-terminal propeptide (P1NP), and C-terminal telopeptide of type I collagen (CTX-1). Analytes were measured using automated methods including the Alinity ci-series (Abbott Laboratories, Chicago, Illinois, USA), and Roche Cobas c501 and c513 analyzers (Roche Diagnostics, Basel, Switzerland).

#### Mental health

Mental well-being was assessed using the 36-item short form survey (SF-36) and the 6-item Subjective Vitality Scale (SVS). SF-36 component scores were calculated using the RAND scoring system. SVS scores were summed to provide an overall vitality rating, with higher scores indicating greater subjective vitality.

#### Exercise adherence

Adherence to the program was calculated based on data from the daily exercise logs. For each participant, the number of sessions completed per exercise category was summed over each 4-week period, averaged across all four exercises, and expressed as a percentage of the 28 possible sessions. These values were then averaged across participants to produce group-level adherence rates for weeks 1–4 and weeks 5–8.

#### Exercise behavior and follow-up engagement

Participants completed brief feedback surveys at 4 and 8 weeks, indicating agreement (via 5-point Likert scale) with statements about perceived strength, fitness, and health improvements. At the 8-week time point, participants were also asked about their intention to continue exercising (1 = continue with same program, 2 = continue independently, 3 = stop, 4 = unsure), and how likely they would be to recommend the program to others (rated from 1 to 10).

To assess longer-term exercise engagement, follow-up surveys were administered at 4 weeks, 3 months, and 12 months post-intervention. Participants reported whether they were currently exercising, the types of activities performed (resistance, cardiovascular, or other), how often they performed each activity (sessions per week), and the typical duration (in minutes) of each session. Total weekly physical activity was calculated by multiplying the reported frequency and session duration for each activity type, then summing across categories for each participant. Group-level values were reported as mean ± SD.

#### Test–retest reliability of the outcome measures

Test–retest reliability for all outcome measures was established in the prior study using a mean-rating (*k* = 2), absolute-agreement, one-way mixed-effects model. Intraclass correlation coefficients (ICC) estimates were interpreted as poor (< 0.5), moderate (0.5 and 0.75), good (0.75 and 0.9) and excellent (> 0.90) (Koo and Li 2016). ICC estimates demonstrated moderate to excellent reliability (0.6–0.99) across outcome measures. Full methodological details of the testing procedures, including protocols for strength, power, endurance, flexibility, and cardiovascular assessments, have been described previously (Kirk et al. 2025).

### Statistical analysis

Data were assessed for normality using the Shapiro–Wilk test and for sphericity using Mauchly’s test. In instances where normality was violated, a Friedman test was used. A one-way repeated-measures analysis of variance was used to analyze changes in the dependent variables over the three timepoints: before the intervention (PRE), after 4 weeks (4-wk), and at the completion of 8 weeks of training (8-wk). In the case of a significant time effect, a Holm’s sequential Bonferroni correction was performed to compare the values between time points (Holm 1979). Eta squared values (*η*^2^) were also reported as a measure of factor variation size, which were interpreted as small (*η*^2^ = 0.01), medium (*η*^2^ = 0.06), and large (*η*^2^ = 0.14) effects (Lakens 2013).

Additionally, changes from PRE to 4-wk were compared to the changes from 4-wk to 8-wk using a linear mixed effects model (LMM). The LMM was specified with the change scores as the dependent variable, the period (1–4 weeks vs. 5–8 weeks) as a fixed effect, and subject as a random effect to account for the repeated measures design and individual variability. This model allowed for the assessment of the intervention effect while controlling for individual variability and potential confounders.

To compare the changes in perceptions between the 4-wk and 8-wk time points, a paired *t* test with Cohen’s d effect sizes were conducted for each perception category (strength, fitness, and health). Cohen’s d values were interpreted as 0.2 for a small effect, 0.5 for a medium effect, and 0.8 for a large effect (Cohen 1988). The significance level was set at *p* ≤ 0.05. All statistical testing was performed using Jamovi version 2.3.26 (Jamovi project, 2018). Data are presented as mean ± standard deviation (SD).

## Results

### Program adherence

Adherence to the home-based exercise program remained high across the 8-week intervention. During weeks 1–4, participants completed 94 ± 11% of sessions. In weeks 5–8, three participants ceased logging their activity after week 5 but verbally confirmed they completed the training. Their continued improvements in physical fitness supported these self-reports. Among those who continued reporting, adherence was 93 ± 11% (range: 18–28 sessions out of 28).

As detailed in Table 1, participants who progressed completed, on average, 14–23 repetitions per set by week 8 in their selected exercises, up from the fixed 10-repetition prescription in weeks 1–4. One participant progressed to the most advanced exercise variations in all categories, performing pistol squats, push-ups, and sit-ups. Others progressed selectively or maintained their initial difficulty while increasing training volume.

Daily steps as measured by FitBit devices did not significantly differ between the control (8321 ± 1051 steps/day), weeks 1–4 (7970 ± 984), and weeks 5–8 (8714 ± 2,906) (*p* = 0.404).

### General health and body composition

As shown in Table 2, no significant changes in body mass, bone mineral density, fat mass, lean mass, resting heart rate systolic or diastolic blood pressure were found over time.

**Table 2:** Body mass, fat mass, % fat, bone mineral content, lean mass, resting heart rate, systolic and diastolic blood pressure, SF-36 and subjective vitality scale scores (mean ± SD, range) before (PRE), after 4 weeks (4-wk) and 8 weeks (8-wk) of the exercise intervention

	PRE	4-wk	8-wk
Body mass (kg)	80.0 ± 14.5 (51.8–105.2)	80.4 ± 14.7 (51.1–105.6)	80.2 ± 14.5 (51.8–105.6)
Fat mass (kg)	30.5 ± 9.7 (13.3–46.0)	30.5 ± 10.2 (13.5–49.0)	30.3 ± 9.5 (13.4–46.7)
Fat (%)	37.2 ± 8.1 (24–46.3)	37.1 ± 8.3 (24.1–46.4)	37.0 ± 7.8 (25.2–45.9)
Bone mineral content (kg)	2.57 ± 0.35 (2.18–3.35)	2.58 ± 0.32 (2.22–3.30)	2.58 ± 0.34 (2.20–3.36)
Lean mass (kg)	47.6 ± 8.8 (37.2–61.4)	47.9 ± 9.1 (36.0–63.0)	48.0 ± 8.8 (37.1–62.4)
Heart rate (bpm)	56.9 ± 6.0 (46–65)	59.5 ± 6.3 (48–68)	59.2 ± 6.3 (48–70)
Systolic blood pressure (mmHg)	120.4 ± 16.6 (100–147)	119.8 ± 17.3 (97–144)	120 ± 15.4 (98–143)
Diastolic blood pressure (mmHg)	69.7 ± 11.0 (53–91)	68.4 ± 10.4 (53–89)	70.4 ± 7.5 (60–87)
SF-36 physical health	53.2 ± 9.7 (37.9–65.2)	49.7 ± 10.9 (22.1–58.1)	48.7 ± 9.4 (28.2–58.8)
SF-36 mental health	39.4 ± 17.1 (3.4–55.2)	51.9 ± 7.5* (35–59.6)	53.2 ± 4.9^*^(42–58.4)
Subjective vitality score	23.9 ± 6.7 (15–35)	29.5 ± 7.4 (20–41)	31.1 ± 6.3 (23–42)

*Significant (*p* < 0.05) difference from PRE

### Physical fitness

**Fig. 1:**
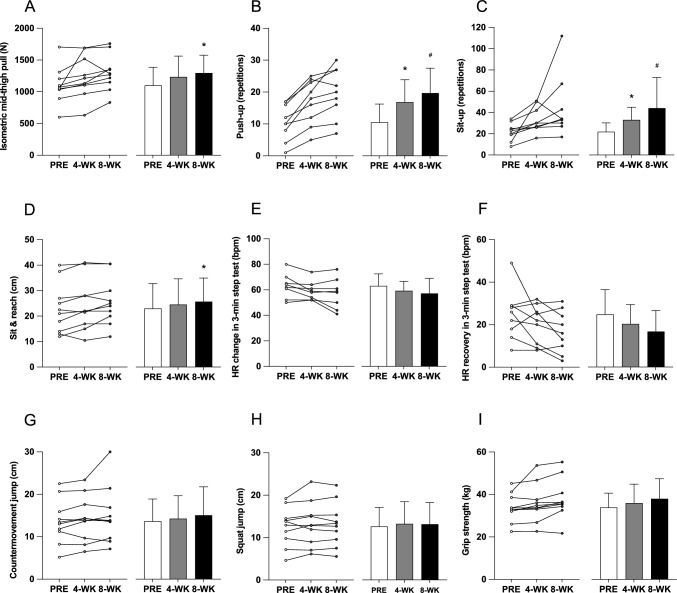
Individual (left) and mean ± SD (right) isometric mid-thigh pull force (**A**), push-up repetitions (**B**), sit-up repetitions (**C**), sit and reach distance (**D**), heart rate change in 3-min step test (**E**), heart rate recovery in the first minute after 3-min step test (**F**), countermovement jump height (**G**), squat jump height (**H**) and grip strength (**I**) before (PRE), after 4 weeks (4-wk) and after 8 weeks of the exercise intervention (8 wk). *Significant (*p* < 0.05) difference from PRE, ^#^Significant (*p* < 0.05) difference from 4 wk

Changes in physical fitness are shown in Fig. 1. No significant changes in grip strength, SJ, or CMJ were detected over time. IMTP force trended upwards over the first four weeks of training from 1101.4 ± 283.7 N at PRE to 1232.9 ± 328.1 N at 4-wk (*p* = 0.112), reaching statistical significance at 8-wk (1296.5 ± 279.3 N, *p* = 0.043, *η*^2^ = 0.076). However, the change from weeks 5 to 8 did not significantly differ from the change observed during weeks 1–4 (*p* = 0.356).

Push-up performance improved from 10.6 ± 5.7 repetitions at PRE to 16.9 ± 7.0 repetitions at 4-wk (*p* = 0.001), and further increased to 19.7 ± 7.8 repetitions at 8-wk (*p* = 0.033 between 4-wk and 8-wk, *η*^2^ = 0.256). The change in push-up performance was greater over weeks 1–4 (5.7 ± 3.3 repetitions) than weeks 5–8 (2.8 ± 3.2 repetitions, *p* = 0.024). Sit-up performance increased from 21.9 ± 8.4 repetitions at PRE to 33.1 ± 11.9 repetitions at 4-wk (*p* = 0.001) then to 44.1 ± 28.9 repetitions at 8-wk (*p* = 0.011 between 4-wk and 8-wk, *η*^2^ = 0.209). The change from weeks 5 to 8 was not significantly different from that of weeks 1 to 4 (*p* = 0.968).

S&R trended upwards over the first four weeks of training from 23.0 ± 9.7 cm at PRE to 24.6 ± 10.1 cm at 4-wk (*p* = 0.093) and reached statistical significance at 8-wk (25.7 ± 9.2 cm, *p* = 0.032, *η*^2^ = 0.014). However, the change from weeks 5 to 8 did not significantly differ from weeks 1 to 4 (*p* = 0.675). No significant changes in the 3-min step test HR (HR_pre_ to HR_post_), HR recovery (HR_pre_ to HR_post,1-min_) or RPE_post_ were observed across timepoints.

### Physical and mental health outcomes

As shown in Table 2, no significant change was observed in the SF-36 physical health component (*p* > 0.05). A significant increase in mental health from 39.4 ± 17.1 at PRE to 51.9 ± 7.5 at 4-wk (*p* = 0.008) was detected. However, no further improvement was observed at 8-wk (53.2 ± 4.9, *p* = 0.767 between 4-wk and 8-wk, *η*^2^ = 0.245), and the change in mental health from weeks 5 to 8 was smaller than during weeks 1 to 4 (*p* = 0.048). No significant change was observed in the subjective vitality score (SVS).

Participants reported that they felt ‘stronger’ (82 ± 12%), ‘fitter’ (78 ± 16%) and ‘healthier’ (80 ± 14%) at 4-wk than baseline. No further improvements were noted between 4 and 8 weeks for perceptions of strength (*p* = 0.195, *d* = − 0.471) or fitness (*p* = 0.681, *d* = − 0.142). However, participants felt ‘healthier’ at 8 weeks than at 4 weeks (91 ± 11%, *p* = 0.013, *d* = − 1.05). ‘Enjoyment’ of the program was 89 ± 10% at 4 weeks and increased to 96 ± 9% at 8 weeks (*p* = 0.013, *d* = − 1.05). At the conclusion of the intervention, 92 ± 8% of participants indicated they would recommend the exercise intervention to a friend or family member. When asked about future exercise intentions, 22% of participants said they would continue the exercise protocol, 67% would exercise in a different way, and 11% were unsure. No participants indicated that they would cease exercising.

### Blood measures

As shown in Table 3, HbA1c exceeded the reference limit in one participant, while HDL, hsCRP, and glucose exceeded the limit in two, one and one participants, respectively, throughout the study. Notably, the variables that were initially outside the reference range in some participants returned within the references range at 4 weeks and remained within the normal range at 8 weeks. This included one participant each for cholesterol, HDL, and hsCRP. A significant decrease in fructosamine from 224.7 ± 28.7 umol/L at PRE to 192.2 ± 33.3 umol/L at 4-wk (*p* = 0.004) was detected with no further change observed at 8 weeks (214.0 ± 25.9 umol/L, *p* = 0.131 between 4 and 8 weeks, *η*^2^ = 0.254). The change in fructosamine from weeks 5 to 8 was smaller than during weeks 1–4 (*p* = 0.001). No significant changes in any of the other blood measures were detected over time.

**Table 3:** Plasma glucose, insulin, HbA1c, triglyceride, total cholesterol, low-density lipoprotein cholesterol (LDL), high-density lipoprotein cholesterol (HDL), high-sensitivity C-reactive protein (hsCRP), procollagen type 1 N-terminal propeptide (P1NP) and C-terminal telopeptide of type I collagen (CTX-1) (mean ± SD, range) before (PRE), after 4 weeks (4-wk) and 8 weeks (8-wk) of exercise

	PRE	4-wk	8-wk	Reference interval
Glucose (mmol/L)	4.9 ± 0.7 (4.1–6.2)	4.8 ± 0.8 (4–6.6)	4.8 ± 0.7 (4–6.3)	3.0–5.4
Insulin (mU/mL)	4.9 ± 0.7 (2.5–12.1)	4.8 ± 0.8 (2.5–6.5)	4.8 ± 0.7 (2.7–12.5)	< 12
HbA1c (mmol/mol)	35.4 ± 5.6 (29.0–47.0)	35.8 ± 4.5 (29.0–44.0)	35.4 ± 5.5 (27.0–47.0)	< 42
Fructosamine (μmol/L)	224.7 ± 28.7 (178.4–280.3)	192.2 ± 33.3* (136.4–234.4)	214.0 ± 25.9 (182.7–277.3)	205–285
Triglyceride (mmol/L)	0.79 ± 0.56 (0.37–2.17)	0.68 ± 0.33 (0.34–1.46)	0.76 ± 0.42 (0.5–1.82)	< 1.7
Total cholesterol (mmol/L)	3.79 ± 1.09 (2.47–5.71)	3.28 ± 0.9 (1.68–4.39)	3.77 ± 0.82 (2.32–4.77)	< 5.5
LDL (mmol/L)	2.53 ± 0.85 (1.14–4.0)	2.12 ± 0.61 (0.84–2.82)	2.41 ± 0.74 (1.16–3.43)	< 3.0
HDL (mmol/L)	1.25 ± 0.38 (0.62–1.70)	1.10 ± 0.36 (0.43–1.58)	1.18 ± 0.32 (0.67–1.68)	> 1.0
hsCRP (mg/L)	1.04 ± 0.67 (0.17–2.08)	0.68 ± 0.42 (0.05–1.37)	0.97 ± 0.70 (0.23–2.17)	< 1.0
P1NP (μg/L)	38.6 ± 14.6 (16.6–57.7)	39.2 ± 14.7 (15.7–60.6)	40.3 ± 16.3 (13.9–62.0)	M: 15–80, F: 15–90
CTX-1 (ng/L)	303.4 ± 142.3 (113.0–564.0)	291.1 ± 138.3 (95.3–547.0)	299.3 ± 149.6 (86.0–555.0)	M: 100–600 F: 150–800 (Menopausal: 50–800)

*Significant (*p* < 0.05) difference from PRE

### Exercise behavior follow-up

At 4 weeks post-intervention, three participants reported continuing the same eccentric-biased daily exercise routine and five participants reported performing either cardiovascular, resistance or yoga-based training, or a combination, 1 to 5 times per week totalling 117 ± 49 min per week. Two participants reported performing no exercise. At 3 months post-intervention, the same three participants continued the eccentric-biased daily exercise routine. Three participants reported performing other types of training 1 to 3 times per week totalling 127 ± 63.5 min per week. Two participants had temporarily ceased exercise due to influenza and a health-related contraindication, respectively. The same two participants who ceased training at 4 weeks post-intervention remained inactive at 3 months.

At the 12-month follow-up, one participant had continued the daily eccentric-biased routine and seven participants were performing either cardiovascular, resistance, or yoga-based training, or a combination, 1 to 4 days per week totalling 124 ± 50 min per week. This group included one participant who had ceased training before the 4-week follow-up and the two participants who had temporarily ceased training before the 3-month follow-up. One participant reported they had ceased regular exercise but mentioned the completion of regular gardening and expressed a desire to recommence training. Finally, the second participant who ceased exercise before the 4-week follow-up remained inactive at 12 months post-intervention. Thus, at 12 months following the intervention, 90% of participants were engaged in some form of regular physical activity.

## Discussion

The present study examined whether extending a minimal-dose, 5-min daily eccentric exercise program from 4 to 8 weeks could sustain or enhance improvements in physical fitness, health markers, and exercise behavior in previously sedentary individuals. The results showed further increases in maximal strength, flexibility, and strength endurance in the period from 4 to 8 weeks above those gained in the first 4 weeks. Additionally, 90% of participants were actively engaged in some form of regular physical activity 12 months after the intervention. These results suggest that a low-dose exercise program can provide substantial health benefit and may effectively encourage sedentary individuals to incorporate exercise into their daily routine. These findings are particularly notable given the extremely low time burden of the intervention (5 min/day) and the absence of direct supervision.

Exercise adherence was high in the first 4 weeks (94%), with almost all sessions completed and tracked. In the second 4-week period, three participants stopped reporting activity after week five, but verbally confirmed that they completed the training. For those who did track the full intervention, adherence remained high (93%) over weeks 5–8. Notably, the recorded exercise data indicated that participants progressively increased their repetitions over time, suggesting that most individuals self-regulated their training volume. This progression likely contributed to the continued improvements in strength and endurance measures observed between weeks 4 and 8, despite the reduced rate of change when compared with the first 4 weeks. At the conclusion of the 8-week program, participants reported ‘feeling’ stronger, fitter, and healthier compared to before exercise commencement. However, while no further improvements were observed in perceptions of strength or fitness between 4 and 8 weeks, participants did report feeling healthier at 8 weeks than at 4 weeks, suggesting that the ongoing participation may have contributed more to overall health perceptions than to additional improvements in strength or fitness perceptions. This aligns with the findings of Steele et al. (2017) who reported that regular regimented resistance training over 6 months significantly improved participants’ perceived abilities to accomplish everyday tasks such as household chores and shopping. Importantly, the results of the present study were achieved after just 8 weeks, illustrating that even a daily 5-min exercise program can significantly enhance perceptions of self-competence and overall physical well-being.

As shown in Fig. 1, while significant improvements in physical fitness measures were observed from baseline, the rate of improvement varied over the study duration. IMTP strength increased by 12% during the first 4 weeks of training and by a further 7% during the second 4 weeks. Although the rate of improvement appeared to slow, the difference in gains between the two periods was not statistically significant. The high variability in individual responses (as indicated by the large standard deviations) suggests that some participants may have continued to make significant gains, while others experienced smaller improvements or plateaus. This variability likely contributed to the non-significant difference between the two periods, indicating that the reduced improvement may reflect differing rates of adaptation among participants rather than a uniform slowdown. Despite this variability, the overall trend of initial rapid gains followed by a slower rate of improvement is consistent with typical strength training adaptations (Kraemer and Ratamess 2004). Importantly, the present study results suggest that the self-selected progression by participants, despite slower gains, was sufficient to sustain continued improvement over the latter 4 weeks of the program.

Similarly, push-up repetitions increased by 104% during the first 4 weeks, with a further 20% increase in the second 4 weeks. This difference was statistically significant (*p* = 0.024), suggesting that while strength gains continued in the latter half of the program, they did so at a slower rate. Sit-up repetitions followed a similar pattern, increasing by 70% during the first 4 weeks and by an additional 29% in the second 4 weeks. However, the large variability in the sit-up results likely contributed to the lack of a statistically significant difference between the two periods. The smaller improvements in weeks 5–8 for both performance measures highlight the potential need for higher training volume or increased intensity to maintain the initial rate of progress (Kraemer and Ratamess 2004). However, these results also emphasize the efficiency of the exercise program, as significant strength gains were achieved with much lower exercise volume and time commitment than traditional regimens (Garber et al. 2011).

Overall, the pattern of rapid initial gains followed by a tapering off is consistent with typical strength training adaptations, where early improvements tend to plateau as participants adapt to the exercises (Kraemer and Ratamess 2004). Despite the slower rate of improvement in weeks 5–8, the program remained effective, with participants continuing to make strength gains during this period. These strength improvements have important implications for enhancing quality of life, including improved performance in daily activities, reduced injury risk (Suchomel et al. 2016), and protection against future disability (Wang et al. 2020). As such, implementing a program like that used in the present study could positively impact health and mitigate issues such as sarcopenia and dynapenia in sedentary populations.

In contrast to the significant improvements observed in IMTP, push-ups, and sit-ups, no changes were observed in grip strength, SJ, or CMJ from 4 to 8 weeks, consistent with findings from our previously published 4-week intervention (Kirk et al. 2025). The lack of improvement in grip strength may be attributed to the absence of grip-specific exercises, as the program did not engage the forearm flexors in a manner necessary to elicit adaptations. Similarly, the failure to improve SJ and CMJ could be due to the lack of high-velocity movement training or explosive intent during the exercise intervention (Kawamori and Newton 2006). These results reinforce that while low-dose exercise protocols can enhance physical capacity, improvements will likely be greatest in tasks that are similar to the exercises performed in training. The movement requirements of an individual should therefore be considered when designing such programs for use outside the research environment.

The sit-and-reach test, assessing flexibility of the lower back and leg musculature (Hartman and Looney 2003), improved by 8% during the first 4 weeks, with a further 7% increase in the second 4 weeks. This is consistent with the findings of Barbosa et al. (2002), who reported a 13% improvement in sit-and-reach distance following 10 weeks of weighted resistance exercise in elderly women. While the direct impact of increased flexibility on movement capacity remains to be fully elucidated, previous research has reported a moderate correlation (*r* = 0.59) between sit-and-reach ability and the functional movement screen (Lockie et al. 2015), suggesting that enhanced flexibility might ease the difficulty of daily activities.

The HR response during the 3 min step test decreased by 5% after the first 4 weeks, followed by a further 4% decrease during the second 4 weeks, resulting in a 10% reduction over the 8-week training program. However, these changes did not reach statistical significance. This contrasts with our previously published 4 week study (Kirk et al. 2025), which included a larger cohort of 22 participants and detected a statistically significant HR reduction of 5% after the first 4 weeks of the same eccentric-biased exercise program. The similar, but non-significant, reduction in the present cohort of 10 participants suggests that the smaller sample size may have limited the ability to detect a significant change, even though the overall percentage decrease in HR was slightly greater. Moreover, the additional 4 weeks of training in the present study did not result in any further statistical change, as the difference in HR reduction between 1–4 weeks and 5–8 weeks was not significant. Therefore, in addition to the smaller sample size, it is also possible that the lack of significant HR change could be attributed to the insufficient intensity and duration of the exercise program to elicit cardiovascular adaptations. This may be due to the nature of eccentric exercises, which require less energy than concentric exercises for the same total work (Douglas et al. 2017; Peñailillo et al. 2014) and therefore may not sufficiently challenge the cardiovascular system to elicit meaningful adaptations. Finally, individual variability in fitness levels and adherence could also have contributed to these results. Overall, this suggests that while the program was effective for strength gains, it was less so for improving cardiovascular fitness.

In the present study, no significant changes in resting HR or systolic or diastolic blood pressure were observed. The lack of significant cardiovascular adaptation may indicate an insufficient intensity and duration of exercise. Previous research supports the proposition that higher intensity and longer duration exercises are necessary to significantly impact cardiovascular fitness and related markers such as blood pressure (Franklin et al. 2022; Kokkinos 2014). Additionally, individual variability in initial blood pressure levels, adherence to the exercise regimen, and overall lifestyle factors such as diet, stress, and sleep quality could have influenced the outcomes.

With respect to lean body mass, no statistically significant change was observed across the group. Although three participants exhibited improvements greater than 1% over the 8 weeks of training, indicating potentially meaningful changes beyond the within-subject variability established in our previously published 4-week intervention (Kirk et al. 2025), these changes were not consistent across the cohort. Overall, the lack of substantial and consistent gains suggests that the exercise regimen may not be of sufficient intensity or duration to achieve significant lean body mass increases for the entire cohort.

The SF-36 survey was used to assess the impacts of the exercise program on physical and mental health outcomes. While no significant (*p* = 0.717) changes were observed in the physical health component, there was a notable 32% improvement in mental health from baseline to 4 weeks (from 39.4 to 51.9) in the present cohort. This improvement, however, did not continue to 8 weeks (53.2 at 8 wk), indicating that most of the mental health gain was achieved during the first 4 weeks. The substantial improvements in the SF-36 scores during the initial 4 weeks suggest that the act of engaging in exercise or the psychological impact of dedicating time to self-care contributes significantly to enhanced mental health outcomes. In contrast, SVS did not change over the study period, although it was observed to improve statistically over 4 weeks in the broader participant group present in our previously published 4 week intervention (Kirk et al. 2025). This discrepancy might be attributed to differences in participant dynamics due to the smaller participant number.

Throughout the 8-week program, a notable decrease in blood fructosamine was observed during the first 4 weeks, reflecting the average levels of blood glucose during the prior 1–3 weeks (Danese et al. 2015). This 17% reduction from baseline, with mean fructosamine levels reaching 192 µmol/L, suggests that the initial phase of the exercise intervention had a significant impact on blood glucose regulation (*p* = 0.001 when compared to the control period). However, the effect was not sustained, as fructosamine levels increased closer to pre-exercise levels by the end of the program (214 µmol/L or a 4% decrease from baseline). Furthermore, the difference in the change scores between weeks 1–4 and weeks 5–8 was statistically significant (*p* = 0.001), indicating a regression toward baseline during the latter half of the program. This rebound in fructosamine suggests that initial improvements in blood glucose regulation may be short-lived without intensification of the program, highlighting the need for progressive overload to maintain metabolic benefits. Importantly, in our previously published 4-week study (Kirk et al. 2025), no significant change in fructosamine was observed, despite using the same intervention and measurement protocol. In that study, the intraclass correlation coefficient (ICC) for fructosamine was 0.67 (95% CI 0.34–0.85), with a coefficient of variation (CV) of 9%, indicating moderate reliability. Given this, the 17% reduction observed in the present study likely reflects a true physiological response beyond typical measurement variability. The lack of sustained improvement and the observed regression in fructosamine levels during weeks 5–8 could indicate that the exercise regimen’s initial positive effect on blood glucose regulation was short-lived, or that other factors, such as unmonitored variations in nutritional intake, played a role. Although participants were instructed not to alter their dietary practices, the absence of dietary monitoring limits our ability to attribute the fluctuations solely to the exercise intervention.

Follow-up data over the subsequent 12 months following the intervention revealed sustained engagement in physical activity among participants. Four weeks after the intervention ended, eight out of 10 participants were still exercising, with three adhering to the eccentric-biased daily routine. By the 3 month mark, six out of 10 participants continued to engage in regular physical activity (with a drop noted due to health issues in two participants). Among these, three participants persisted with the eccentric-biased daily routine, while the other three had initiated various forms of training including strength, cardiovascular, yoga, or dance exercise, averaging 127 min per week. This not only surpassed the weekly total of ~ 35 min (5 daily min × 7 days per week) achieved in the eccentric exercise routine but also approached the WHO’s recommended 150 min of moderate-intensity exercise per week (Bull et al. 2020). By the 12-month follow-up, nine of the 10 participants were actively engaged in some form of regular physical activity. One individual continued with the daily eccentric routine and seven participants were involved in diverse forms of exercise training 1 to 4 days per week, totalling an average of 124 min per week. This group included individuals who had previously paused their training due to temporary health issues at the 3-month mark, demonstrating a resumption of physical exercise after the interruptions. Finally, one participant reported participating in regular gardening most days of the week. The consistency of activity at the 3- and 12-month marks indicates the maintenance of exercise habits yet points to the potential need for additional interventions to allow the individuals to consistently meet or exceed WHO guidelines.

Interestingly, participants’ self-reported intentions at the end of the 8-week program closely aligned with their behavior over the following 12 months, suggesting that the act of public commitment and goal-setting may have played a meaningful role in sustaining engagement (Matthews and Kraus 2007; Klein et al. 2020). While long-term adherence rates in other interventions have ranged from 57% in fitness club members (Gjestvang et al. 2020) to approximately 84% in a combined facility- and home-based program with structured supervision (Cadmus-Bertram et al. 2014), the present study achieved comparable outcomes without ongoing contact or structured support, further reinforcing the feasibility of this minimal-dose, home-based eccentric exercise model.

These results are promising for real-world application, demonstrating that a simple, minimalistic exercise program can instill long-lasting exercise habits in previously sedentary individuals, potentially reducing the risk of lifestyle-related diseases. Moreover, the high adherence rate to regular exercise over the 12 month follow-up highlights the program’s potential to foster long-term behavioral change. To optimize health benefits, however, further support and program modifications may be required to ensure participants achieve and maintain the physical activity level recommended by the WHO (Bull et al. 2020).

One important limitation of the present study was its limited statistical power, which may have influenced the ability to detect significant changes. It was hypothesized that extending the exercise program by 4 weeks would result in sustained improvements across outcome measures, with certain measures like resting blood pressure expected to reach statistical significance at 8 weeks, based on the near-significant changes observed at 4 weeks in our previously published study (Kirk et al. 2025). However, not all outcomes aligned with this expectation, particularly the lack of significant HR change in the 3-min step test, which had previously reached significance following the initial 4-week intervention (Kirk et al. 2025) but was not replicated here. This discrepancy may be attributed to the smaller sample size and reduced statistical power in the current study. While most participants logged each daily session completed, including the exercises performed, repetitions, and RPE, we could not directly verify the duration or strict adherence to the ~ 5 min daily protocol. It is therefore possible that some participants exceeded the prescribed dose, which may partly explain variability in the observed outcomes.

In addition, no physiological or health outcomes were measured beyond the 8-week intervention period, and thus, whether continued physical activity engagement had a positive impact on health or fitness could not be determined. Follow-up testing was considered but ultimately deemed impractical due to the lack of standardized exercise exposure after the intervention, which would have limited the meaningful interpretation of any physical outcome data. The study cohort also represented a self-selected subset of participants from a prior 4-week study who volunteered to continue, potentially introducing a motivational bias. However, follow-up data from the initial 4-week intervention (Kirk et al. 2025) showed that 83% of participants also reported continued physical activity 4 weeks after the intervention, suggesting that ongoing engagement was common and not limited to the self-selected group in the present study. Despite these limitations, the study offers valuable insight into the potential of minimalistic exercise programs to promote long-term adherence and improve physical health in sedentary individuals, and may inform more scalable or sophisticated models in future research.

In conclusion, the 5-min home-based eccentric exercise program significantly improved muscle strength, flexibility, and mental health over an 8-week period with high adherence in healthy but sedentary individuals. Importantly, while the initial 4 weeks of the program yielded substantial improvements, the gains observed during the second 4-week phase were generally smaller, indicating a tapering off in the rate of improvement. This suggests that the most significant benefits were achieved early in the intervention, with continued but reduced progress in the latter phase. Nevertheless, nine of the 10 participants continued to engage in regular exercise or physical activity beyond the intervention, highlighting the potential of this minimalistic approach to instill lasting exercise habits. This sustained engagement illustrates the program’s effectiveness in transitioning sedentary individuals to more active lifestyles. Such interventions could be crucial in public health strategies aimed at mitigating the risks associated with sedentary behavior by providing an accessible and sustainable gateway to regular physical activity.

## Supplementary Information

Below is the link to the electronic supplementary material.

Supplementary file1 (PDF 2205 KB)

## Data Availability

All data generated or analysed during this study are included in this published article.

## Abbreviations

1-RM: One-repetition maximum
CMJ: Countermovement jump
CTX-1: C-terminal telopeptide of type I collagen
DBP: Diastolic blood pressure
DEXA: Dual-energy X-ray absorptiometry
DPB: Diastolic blood pressure
EDTA: Ethylenediaminetetraacetic acid
HbA1c: Hemoglobin A1c
HDL: High-density lipoprotein
HR: Heart rate
HR_pre_: Pre-exercise heart rate
HR_post_: Post-exercise heart rate
HR_post,1-min_: Heart rate at 1-min post-exercise
hsCRP: High-sensitivity C-reactive protein
IMTP: Isometric mid-thigh pull
LMM: Linear mixed effects model
LDL: Low-density lipoprotein
P1NP: Procollagen type 1 N-terminal propeptide
PPT: Plasma preparation tube
RPE: Rating of perceived exertion
SBP: Systolic blood pressure
SF-36: 36-Item short form survey
SJ: Squat jump
SST: Serum separating tubes
SVS: Subjective vitality scale
S&R: Sit-and-reach test
V-Up: An abdominal exercise
WHO: World Health Organization
wk: Week

## References

[CR1] Australian Institute of Health and Welfare (2023) Physical activity. AIHW, Canberra

[CR2] Barbosa AR, Santarém JM, Jacob Filho W, Marucci MFN (2002) Effects of resistance training on the sit-and-reach test in elderly women. J Strength Cond Res 16(1):14–18 11834101

[CR3] Bennie JA, Pedisic Z, Timperio A, Crawford D, Dunstan D, Bauman A, Van Uffelen J, Salmon J (2015) Total and domain-specific sitting time among employees in desk-based work settings in Australia. Aust N Z J Public Health 39(3):237–242 25545803 10.1111/1753-6405.12293

[CR4] Bowles HR, Morrow JR Jr, Leonard BL, Hawkins M, Couzelis PM (2002) The association between physical activity behavior and commonly reported barriers in a worksite population. Res Q Exerc Sport 73(4):464–470 12495249 10.1080/02701367.2002.10609047

[CR5] Bull FC, Al-Ansari SS, Biddle S, Borodulin K, Buman MP, Cardon G, Carty C, Chaput J-P, Chastin S, Chou R (2020) World Health Organization 2020 guidelines on physical activity and sedentary behaviour. Br J Sports Med 54(24):1451–1462 33239350 10.1136/bjsports-2020-102955 PMC7719906

[CR6] Cadmus-Bertram L, Irwin M, Alfano C, Campbell K, Duggan C, Foster-Schubert K, Wang C-Y, McTiernan A (2014) Predicting adherence of adults to a 12-month exercise intervention. J Phys Act Health 11(7):1304–1312 24176780 10.1123/jpah.2012-0258 PMC5972545

[CR7] Cohen J (1988) Statistical power analysis for the behavioral sciences. Routledge, New York (NY)

[CR8] Danese E, Montagnana M, Nouvenne A, Lippi G (2015) Advantages and pitfalls of fructosamine and glycated albumin in the diagnosis and treatment of diabetes. J Diabetes Sci Technol 9(2):169–176 25591856 10.1177/1932296814567227 PMC4604592

[CR9] Diong J, Carden PC, O’Sullivan K, Sherrington C, Reed DS (2022) Eccentric exercise improves joint flexibility in adults: a systematic review update and meta-analysis. Musculoskelet Sci Pract 60:102556 35390669 10.1016/j.msksp.2022.102556

[CR10] Dishman RK, Buckworth J (2013) Adherence to physical activity. Physical activity and mental health. Taylor & Francis, Milton Park, pp 63–80

[CR11] Douglas J, Pearson S, Ross A, McGuigan M (2017) Eccentric exercise: physiological characteristics and acute responses. Sports Med 47:663–675 27638040 10.1007/s40279-016-0624-8

[CR12] Franklin BA, Eijsvogels TM, Pandey A, Quindry J, Toth PP (2022) Physical activity, cardiorespiratory fitness, and cardiovascular health: A clinical practice statement of the American Society for Preventive Cardiology Part II: physical activity, cardiorespiratory fitness, minimum and goal intensities for exercise training, prescriptive methods, and special patient populations. Am J Prev Cardiol 12:100425 36281325 10.1016/j.ajpc.2022.100425 PMC9586849

[CR13] Garber CE, Blissmer B, Deschenes MR, Franklin BA, Lamonte MJ, Lee I-M, Nieman DC, Swain DP (2011) American college of sports medicine position stand. Quantity and quality of exercise for developing and maintaining cardiorespiratory, musculoskeletal, and neuromotor fitness in apparently healthy adults: guidance for prescribing exercise. Med Sci Sports Exerc 43(7):1334–1359 21694556 10.1249/MSS.0b013e318213fefb

[CR14] Gjestvang C, Abrahamsen F, Stensrud T, Haakstad LA (2020) Motives and barriers to initiation and sustained exercise adherence in a fitness club setting - a one-year follow-up study. Scand J Med Sci Sports 30(9):1796–1805 32488898 10.1111/sms.13736 PMC7497044

[CR15] Hartman JG, Looney M (2003) Norm-referenced and criterion-referenced reliability and validity of the back-saver sit-and-reach. Meas Phys Educ Exerc Sci 7(2):71–87

[CR16] Hoare E, Stavreski B, Jennings GL, Kingwell BA (2017) Exploring motivation and barriers to physical activity among active and inactive Australian adults. Sports 5(3):47 29910407 10.3390/sports5030047 PMC5968958

[CR17] Hollander DB, Durand RJ, Trynicki JL, Larock D, Castracane VD, Hebert EP, Kraemer RR (2003) RPE, pain, and physiological adjustment to concentric and eccentric contractions. Med Sci Sports Exerc 35(6):1017–1025 12783051 10.1249/01.MSS.0000069749.13258.4E

[CR18] Holm S (1979) A simple sequentially rejective multiple test procedure. Scandinavian journal of statistics. Wiley, Boca raton, pp 65–70

[CR19] Hughes DC, Ellefsen S, Baar K (2018) Adaptations to endurance and strength training. Cold Spring Harb Perspect Med 8(6):a029769 28490537 10.1101/cshperspect.a029769 PMC5983157

[CR20] Katsura Y, Takeda N, Hara T, Takahashi S, Nosaka K (2019) Comparison between eccentric and concentric resistance exercise training without equipment for changes in muscle strength and functional fitness of older adults. Eur J Appl Physiol 119(7):1581–1590 31055678 10.1007/s00421-019-04147-0

[CR21] Kawamori N, Newton RU (2006) Velocity specificity of resistance training: actual movement velocity versus intention to move explosively. Strength Cond J 28(2):86–91

[CR22] Kay AD, Baxter B, Hill M, Blazevich A (2023) Effects of eccentric resistance training on lower-limb passive joint range of motion: a systematic review and meta-analysis. Med Sci Sports Exerc 55(4):710–721 36730587 10.1249/MSS.0000000000003085

[CR23] Kirk BJC, Mavropalias G, Blazevich AJ, Cochrane Wilkie JL, Molan A, Nosaka K (2025) Effects of a daily, home-based, 5-minute eccentric exercise program on physical fitness, body composition, and health in sedentary individuals. Eur J Appl Physiol. 10.1007/s00421-025-05757-7 40131475 10.1007/s00421-025-05757-7 PMC12354585

[CR24] Klein HJ, Lount RB Jr, Park HM, Linford BJ (2020) When goals are known: the effects of audience relative status on goal commitment and performance. J Appl Psychol 105(4):372 31414830 10.1037/apl0000441

[CR25] Kokkinos P (2014) Cardiorespiratory fitness, exercise, and blood pressure. Hypertension 64(6):1160–1164 25245388 10.1161/HYPERTENSIONAHA.114.03616

[CR26] Koo TK, Li MY (2016) A guideline of selecting and reporting intraclass correlation coefficients for reliability research. J Chiropr Med 15(2):155–163 27330520 10.1016/j.jcm.2016.02.012 PMC4913118

[CR27] Kraemer WJ, Ratamess NA (2004) Fundamentals of resistance training: progression and exercise prescription. Med Sci Sports Exerc 36(4):674–688 15064596 10.1249/01.mss.0000121945.36635.61

[CR28] Lakens D (2013) Calculating and reporting effect sizes to facilitate cumulative science: a practical primer for t-tests and ANOVAs. Front Psychol 4:863 24324449 10.3389/fpsyg.2013.00863 PMC3840331

[CR29] Lee I-M, Shiroma EJ, Lobelo F, Puska P, Blair SN, Katzmarzyk PT (2012) Effect of physical inactivity on major non-communicable diseases worldwide: an analysis of burden of disease and life expectancy. Lancet 380(9838):219–229 22818936 10.1016/S0140-6736(12)61031-9 PMC3645500

[CR30] Lockie RG, Schultz AB, Callaghan SJ, Jordan CA, Luczo TM, Jeffriess MD (2015) A preliminary investigation into the relationship between functional movement screen scores and athletic physical performance in female team sport athletes. Biol Sport 32(1):41–51 25729149 10.5604/20831862.1127281 PMC4314603

[CR31] Mahindru A, Patil P, Agrawal V (2023) Role of physical activity on mental health and well-being: a review. Cureus. 10.7759/cureus.33475 36756008 10.7759/cureus.33475 PMC9902068

[CR32] Marcus BH, Forsyth LH, Stone EJ, Dubbert PM, McKenzie TL, Dunn AL, Blair SN (2000) Physical activity behavior change: issues in adoption and maintenance. Health Psychol 19(1S):32 10709946 10.1037/0278-6133.19.suppl1.32

[CR33] Matthews G, Kraus S (2007) The impact of commitment, accountability, and written goals on goal achievement. Psychology, vol 3. Department of Psychology, Dominican University of California, Faculty Presentations, San Rafael

[CR34] Miller PC, Hall EE, Chmelo EA, Morrison JM, DeWitt RE, Kostura CM (2009) The influence of muscle action on heart rate, RPE, and affective responses after upper-body resistance exercise. J Strength Cond Res 23(2):366–372 19204573 10.1519/JSC.0b013e31818548f6

[CR35] Morgan AJ, Parker AG, Alvarez-Jimenez M, Jorm AF (2013) Exercise and mental health: an exercise and sports science Australia commissioned review. J Exerc Physiol 16(4):64–73

[CR36] Nuzzo JL, Pinto MD, Kirk BJC, Nosaka K (2024) Resistance exercise minimal dose strategies for increasing muscle strength in the general population: an overview. Sports Med 54(5):1139–1162 38509414 10.1007/s40279-024-02009-0 PMC11127831

[CR37] Peñailillo L, Blazevich A, Nosaka K (2014) Energy expenditure and substrate oxidation during and after eccentric cycling. Eur J Appl Physiol 114:805–814 24390692 10.1007/s00421-013-2816-3

[CR38] Roig M, O’Brien K, Kirk G, Murray R, McKinnon P, Shadgan B, Reid W (2009) The effects of eccentric versus concentric resistance training on muscle strength and mass in healthy adults: a systematic review with meta-analysis. Br J Sports Med 43(8):556–568 18981046 10.1136/bjsm.2008.051417

[CR39] Schoenfeld BJ, Ogborn DI, Vigotsky AD, Franchi MV, Krieger JW (2017) Hypertrophic effects of concentric vs. eccentric muscle actions: a systematic review and meta-analysis. J Strength Cond Res 31(9):2599–2608 28486337 10.1519/JSC.0000000000001983

[CR40] Steele J, Raubold K, Kemmler W, Fisher J, Gentil P, Giessing J (2017) The effects of 6 months of progressive high effort resistance training methods upon strength, body composition, function, and wellbeing of elderly adults. BioMed Res Int 2017:2541090 28676855 10.1155/2017/2541090 PMC5476889

[CR41] Suchomel TJ, Nimphius S, Stone MH (2016) The importance of muscular strength in athletic performance. Sports Med 46:1419–1449 26838985 10.1007/s40279-016-0486-0

[CR42] Vallejo AF, Schroeder ET, Zheng L, Jensky NE, Sattler FR (2006) Cardiopulmonary responses to eccentric and concentric resistance exercise in older adults. Age Ageing 35(3):291–297 16638770 10.1093/ageing/afj082

[CR43] Wang DX, Yao J, Zirek Y, Reijnierse EM, Maier AB (2020) Muscle mass, strength, and physical performance predicting activities of daily living: a meta-analysis. J Cachexia Sarcopenia Muscle 11(1):3–25 31788969 10.1002/jcsm.12502 PMC7015244

[CR44] Yoshida R, Kasahara K, Murakami Y, Sato S, Tanaka M, Nosaka K, Nakamura M (2024) Weekly minimum frequency of one maximal eccentric contraction to increase muscle strength of the elbow flexors. Eur J Appl Physiol 124(1):329–339 37505230 10.1007/s00421-023-05281-6

