# Supplementary material for: Effects of an 8-week minimal-dose home-based eccentric exercise program on physical health and exercise adherence
Source: Eur J Appl Physiol. 2025 Oct 9;126(3):1671–84. doi: 10.1007/s00421-025-05989-7 (PMC13013343; doi:10.1007/s00421-025-05989-7)
Supplement: Supplementary file 1 — Supplementary file1 (PDF 2205 KB) [file 421_2025_5989_MOESM1_ESM.pdf]

LEGS

Chair Squat

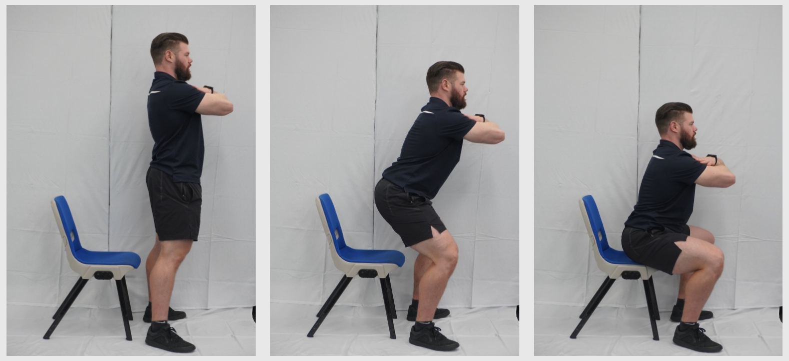

Start *Middle* *Finish*

Sit down slowly to a chair over 5 seconds from a standing position. Stand up to return to start position.

One-Leg Biased Chair Squat

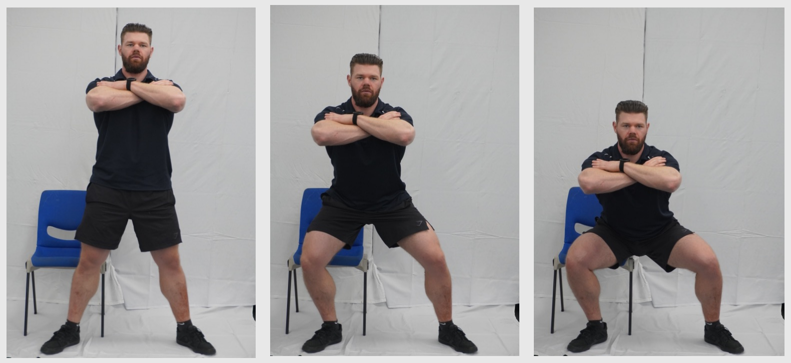

Start *Middle* *Finish*

Sit down slowly to a chair over 5 seconds distributing your weight through the leg nearest the chair.

Pistol Squat

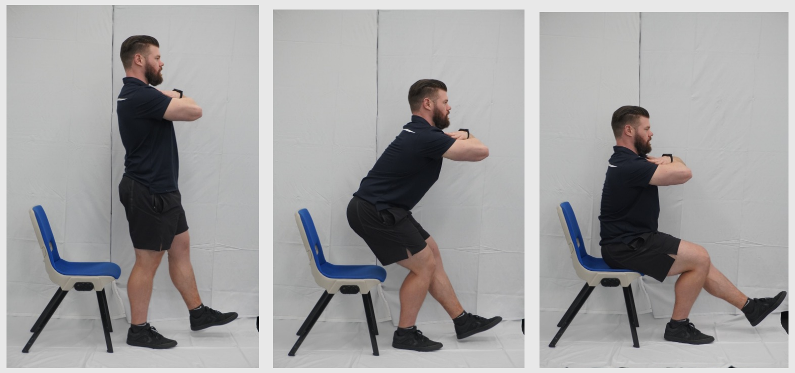

Start *Middle* *Finish*

With one leg off the ground. Sit down slowly to a chair over 5 seconds from a standing position

CHEST

Wall Push-Up

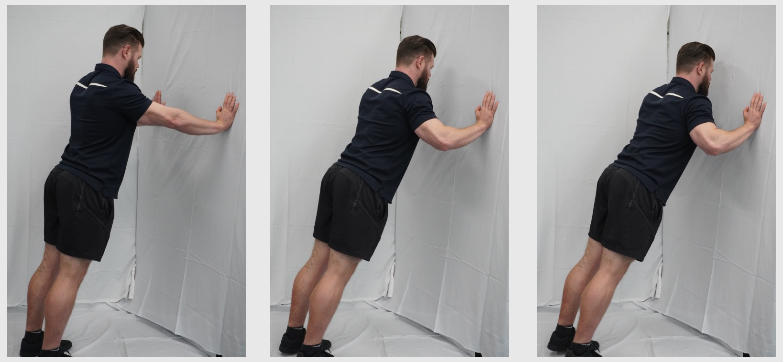

Start *Middle* *Finish*

Descend over 5 seconds toward the wall by pulling the elbows behind the body until the head reaches the wall

One Arm Wall Push-Up

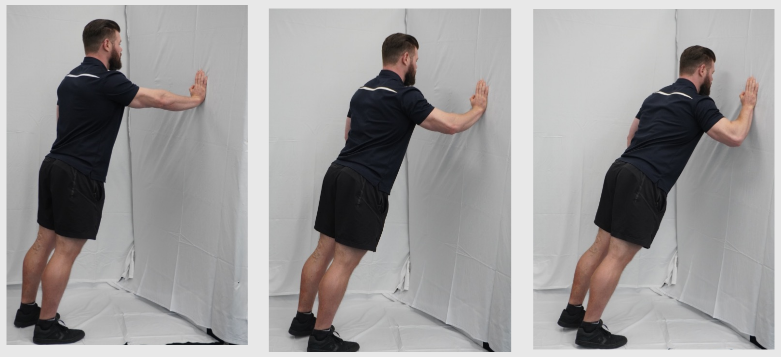

Start *Middle* *Finish*

With one arm on a wall, lower toward the wall over 5 seconds by pulling the elbow back until the head reaches the wall.

Table Push-Up

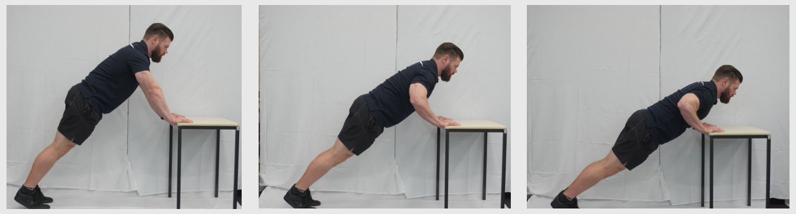

Start *Middle* *Finish*

Place hands on a table with the legs and torso at ~45°. Descend over 5 seconds toward the table by pulling the elbows behind the body until the elbow reaches $\leq 90°$, ensuring range of motion is consistent between repetitions

Knee Push-Up

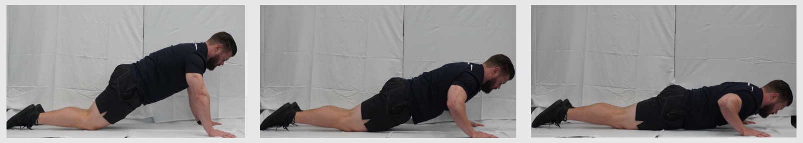

Start *Middle* *Finish*

With hands at shoulder width and knees planted on the floor, descend over 5 seconds toward the floor by pulling the elbows behind the body until the elbow reaches $\leq 90°$ ensuring at the bottom that the head is forward of the hands in the sagittal plane.

Push-Up

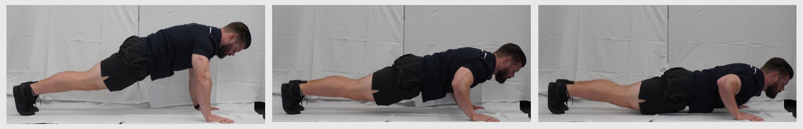

Start *Middle* *Finish*

With hands at shoulder width and feet planted on the floor, descend over 5 seconds toward the floor by pulling the elbows behind the body until the elbow reaches $\leq 90°$ ensuring at the bottom that the head is forward of the hands in the sagittal plane.

ABDOMINALS

Chair Recline Back

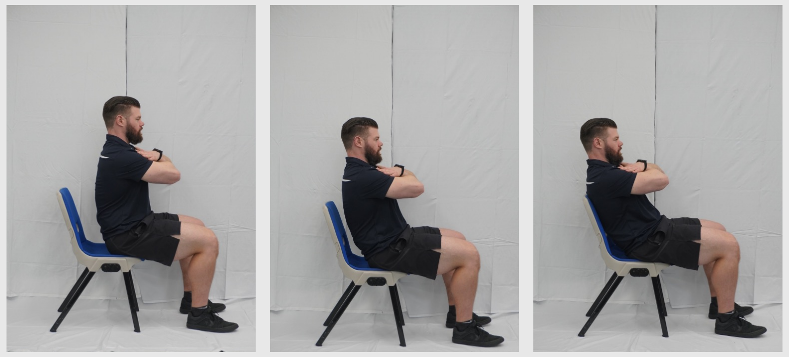

Start *Middle* *Finish*

Sit on the front of a chair and recline to the back of the chair over 5 seconds. Sit up to return to the start position.

Chair Recline Back; Legs Straight

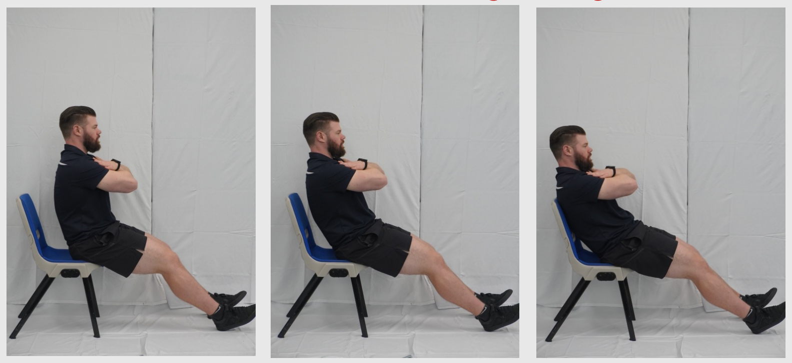

Start *Middle* *Finish*

Sit on the front of a chair with legs straight and recline to the back of the chair over 5 seconds. Sit up to return to the start position.

Sit-Up

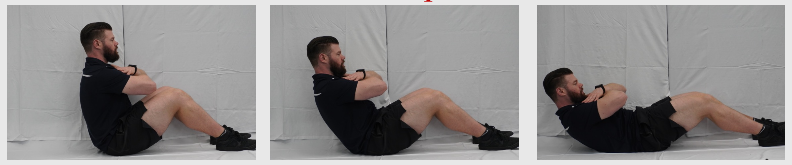

Start *Middle* *Finish*

While seated on the floor, slowly recline over 5 seconds until the mid-back touches the floor. Sit up to return to the start position.

V-Up

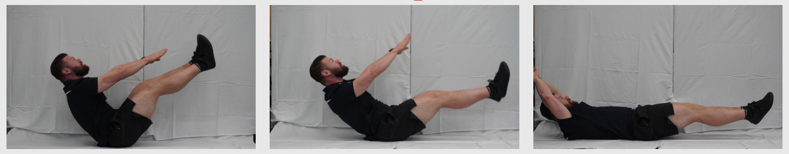

Start *Middle* *Finish*

Lying supine with legs extended and arms overhead. Simultaneously lift the legs and upper body to form a V-shape, then lower back down over 5 seconds

CALVES

Heel Drop

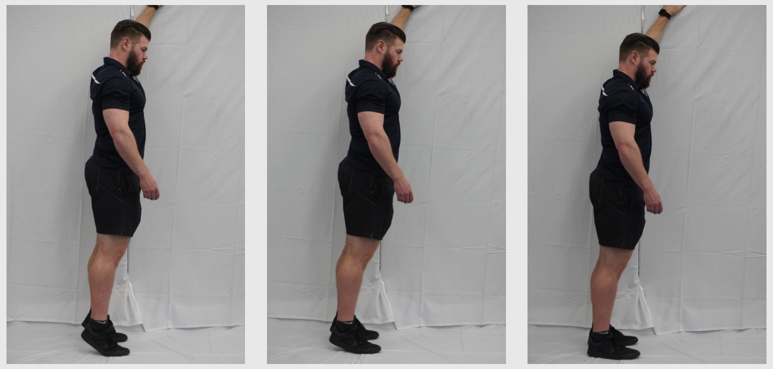

Start *Middle* *Finish*

Raise the heels and lower the heels over 5 seconds

Heel Drop Overstretch (2 Feet Up, 1 Down)

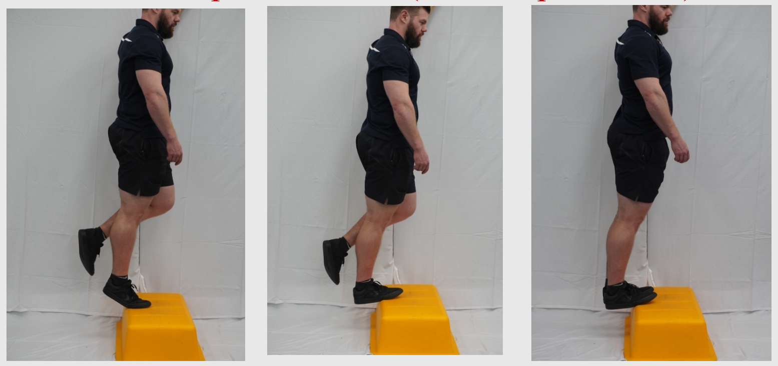

Start *Middle* *Finish*

Raise the heels, then lower on one foot over 5 seconds. Return to start position using both feet

One Leg Heel Drop Overstretch (1 Leg)

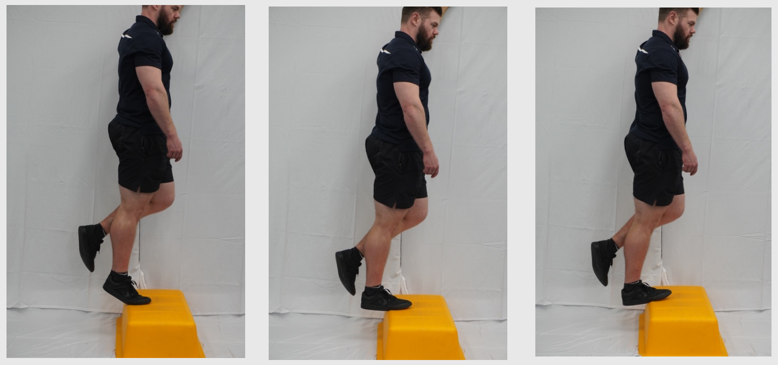

Start *Middle* *Finish*

On one leg, raise the heel, then lower the heels over 5 seconds. Return to start position using one leg
